# Daily Sugar-Sweetened Beverage Consumption, by Disability Status, Among Adults in 23 States and the District of Columbia

**DOI:** 10.5888/pcd14.160606

**Published:** 2017-12-14

**Authors:** Sunkyung Kim, Sohyun Park, Dianna D. Carroll, Catherine A. Okoro

**Affiliations:** 1 Northrop Grumman, Atlanta, Georgia; 2 Division of Nutrition, Physical Activity, and Obesity, National Center for Chronic Disease Prevention and Health Promotion, Centers for Disease Control and Prevention, Atlanta, Georgia; 3 Division of Human Development and Disability, National Center on Birth Defects and Developmental Disabilities, Centers for Disease Control and Prevention, Atlanta, Georgia; 4 Commissioned Corps Officer, US Public Health Service, Atlanta, Georgia; 5 Population Health Surveillance Branch, Division of Population Health, National Center for Chronic Disease Prevention and Health Promotion, Centers for Disease Control and Prevention, Atlanta, Georgia

## Abstract

**Introduction:**

Information on dietary intake, including sugar-sweetened beverages (SSBs), for adults with disabilities is limited. Such information can inform interventions to prevent chronic disease and promote health among adults with disabilities. The objective of this study was to describe the associations between SSB consumption and disability among adults.

**Methods:**

We examined data on adults aged 18 years or older in 23 states and the District of Columbia who participated in the 2013 Behavioral Risk Factor Surveillance System (n = 150,760). Participants who reported a limitation in any activity caused by physical, mental, or emotional problems or who reported use of special equipment were considered to have a disability (n = 41,199). Participants were classified as daily SSB consumers (≥1 time/d) and non-daily SSB consumers (<1 time/d). Multivariable logistic regression was used to examine associations between daily SSB intake and disability after controlling for sociodemographic characteristics. An interaction effect between disability and obesity status was tested to consider obesity status as a potential effect modifier.

**Results:**

The prevalence of drinking SSBs at least once daily was significantly higher among adults with disabilities (30.3%) than among adults without disabilities (28.6%) (*P* = .01). After controlling for sociodemographic characteristics, among nonobese adults, the odds of daily SSB intake were significantly higher among adults with disabilities than among adults without disabilities (adjusted odds ratio = 1.27, *P* < .001). Among obese adults, daily SSB intake was not associated with disability status (adjusted odds ratio = 0.97; *P* = .58).

**Conclusion:**

Our findings highlight the need for increased awareness of SSB consumption among adults with disabilities.

## Introduction

Various definitions of disability exist. The US Census Bureau defines a disability as an impairment or limitation in any activities caused by communicative, mental, or physical problems (1). Disabilities affect more than 56 million people in the United States, and the prevalence increases with increases in age (1). In 2010, 21.3% of people aged 15 years or older had a disability, whereas 49.8% of older adults (≥65 y) had a disability (1). Additionally, more than half of people with a disability had a severe disability in 2010, and disability-associated health care expenditures were estimated to be $400 billion among US adults in 2006 (1,2).

Eating a healthy diet is an important lifestyle behavior that contributes to overall health and nutritional well-being. However, people with disabilities may be at increased risk of nutritional deficiency, because barriers to eating a healthy diet often are biopsychosocial (eg, underlying physical or mental disease, loss of appetite, social isolation) (3,4). Adults with moderate to severe disabilities (eg, those who cannot walk independently, those who have significant cognitive limitations) may not be able to choose their foods independently, be able to cook for themselves, or have access barriers to healthy affordable food outlets. Instead, people with disabilities may consume processed foods or fast foods that have limited nutritional value more frequently than those without disabilities. These behaviors may negatively affect their health. The prevalence of obesity is significantly higher among adults with disabilities than among those without, and adults with disabilities are more likely to have risk factors for chronic diseases (5,6).

Sugar-sweetened beverages (SSBs) are defined as “liquids that are sweetened with various forms of added sugars. These beverages include, but are not limited to, soda (regular, not sugar-free), fruitades, sports drinks, energy drinks, sweetened waters, and coffee and tea beverages with added sugars” by the 2015–2020 Dietary Guidelines for Americans (7) and are a significant source of added sugars and energy in the diet of US adults (8). According to the 2011–2014 National Health and Nutrition Examination Survey (NHANES), approximately half of US adults drank SSBs on a given day and mean daily energy intake from SSBs was 145 kcal (9). SSBs provide calories with little or no nutritional value and are associated with increased health risks, including weight gain and obesity, cardiovascular disease, kidney disease, asthma, and type 2 diabetes as well as poor diet quality, physical inactivity, and smoking (10–16).

One study reported that older adults (≥65 y) with disabilities were less likely to have a healthy weight and engage in physical activity, but it also reported no difference in fruit and vegetable intake between older adults with disabilities and older adults without disabilities (17). Although general information on dietary intake among adults with disabilities is available (18), no details are available on the association between disability status and SSB consumption. To the best of our knowledge, our study is the first to investigate this topic.

Habitual SSB consumption among adults with disabilities can be more problematic than among adults without disabilities because the excess sugar intake, combined with limited physical activity, can expedite weight gain and increase the risk of chronic diseases. The prevalence of disability is expected to increase as the population ages, so a better understanding of SSB consumption among adults with disabilities could help in designing interventions and targeting messages about healthy dietary choices and further help to prevent chronic diseases among people with disabilities. The objectives of this study were to estimate the prevalence of daily SSB consumption by disability status and sociodemographic characteristics among US adults and to describe associations between SSB consumption and disability status while considering obesity status as a potential effect modifier.

## Methods

This cross-sectional study used data from the 2013 Behavioral Risk Factor Surveillance System (BRFSS) from 23 states and the District of Columbia. The BRFSS is the largest ongoing random-digit–dialed telephone health survey in the world; it is conducted via both landline and cellular telephones. For the landline telephone survey, data are collected from a randomly selected adult in a household. For the cellular telephone version, data are collected from an adult who participates by using a cellular telephone. It is a cross-sectional and state-based system of health surveys established in 1984 by the Centers for Disease Control and Prevention (CDC) (19). BRFSS surveys a representative sample of community-dwelling adults (aged ≥18 y) in all 50 states, the District of Columbia, and the US territories Guam and Puerto Rico to obtain information on health risk and behaviors, health practices for preventing disease, and health care access primarily related to chronic disease, injury, and death. In 2013, 23 states (Alaska, Arizona, California, Connecticut, Indiana, Iowa, Kansas, Kentucky, Louisiana, Maryland, Minnesota, Mississippi, Nebraska, New Jersey, New York, North Carolina, Ohio, Oklahoma, South Carolina, Utah, Vermont, West Virginia, and Wisconsin) and the District of Columbia administered an optional module on sugar drinks. A total of 161,317 adults completed the optional module. For this analysis, participants with missing data on SSB intake (n = 2,471), disability status (n = 663), or self-reported weight or height (n = 7,423) were excluded, which resulted in a final analytic sample of 150,760 adults. The BRFSS has been reviewed by the Human Research Protection Office of CDC and determined to be exempt research.

### Variables

The outcome variable was frequency of daily SSB consumption. The BRFSS module on sugar drinks consisted of 2 questions related to SSB intake: “During the past 30 days, how often did you drink regular soda or pop that contains sugar? Do not include diet soda or diet pop.” and “During the past 30 days, how often did you drink sugar-sweetened fruit drinks (such as Kool-Aid and lemonade), sweet tea, and sports or energy drinks (such as Gatorade and Red Bull)? Do not include 100% fruit juice, diet drinks, or artificially sweetened drinks.” For each question, respondents answered the number of times per day, per week, or per month they consumed a SSB. To convert frequency of weekly and monthly intake to frequency of daily intake, weekly frequency was divided by 7 and monthly frequency was divided by 30. The frequency of daily SSB intake was then calculated as the sum of the number of times daily that soda, fruit drink, sweet tea, and sports or energy drink were consumed. The participants were classified as daily SSB consumers (≥1 time/d) and non-daily SSB consumers (<1 time/d), which was used as the main outcome. At least once daily was used to define habitual SSB consumers (ie, daily intake) and was based on clinical research that showed increased risk for coronary heart disease and stroke with daily SSB intake (20,21).

The main exposure variable was disability status (yes or no). Those who reported a limitation in any activities due to physical, mental, or emotional problems or who reported special equipment use were defined as having a disability. Adults with disabilities were identified based on a response of yes to either of 2 questions: “Are you limited in any way in any activities because of physical, mental, or emotional problems?” and “Do you now have any health problem that requires you to use special equipment, such as a cane, a wheelchair, a special bed, or a special telephone?” Participants who answered no to both questions were considered to have no disability.

Covariates were the following sociodemographic factors: sex, age (18−24 y, 25−34 y, 35−44 y, 45−54 y, 55−64 y, and ≥65 y), race/ethnicity (non-Hispanic white, non-Hispanic black, non-Hispanic other, or Hispanic), annual household income (<$25,000, $25,000 to <$50,000, $50,000 to <$75,000, ≥$75,000, and unknown), educational attainment (<high school diploma, high school diploma, and >high school diploma), marital status (married/couple, previously married, and never married). Body mass index (BMI, kg/m^2^), calculated by using self-reported data on height and weight, was used to dichotomize respondents into 2 groups: nonobese (BMI <30) and obese (BMI ≥30).

### Statistical analysis

We calculated the unadjusted prevalence of daily SSB consumption, by disability status and sociodemographic variables, and assessed the differences in prevalence of daily SSB consumption between adults with disability and without disability for each demographic subgroup by using the χ^2^ test. To examine the adjusted association between SSB consumption and disability, we applied a multivariable logistic regression model to calculate adjusted odds ratios (ORs) and 95% confidence intervals (CIs). In the analysis, daily SSB consumption was treated as a binary outcome and all covariates were treated as categorical variables. Because previous studies showed significant associations between SSB consumption and obesity (17) and associations between disability and obesity (5), we tested for an interaction between disability and obesity status to consider obesity status as a potential effect modifier on the association between SSB consumption and disability in the multivariable regression. Because we found a significant interaction between disability and obesity status (*P* < .001), we tabulated the data on the association of SSB intake and disability by obesity status.

We considered *P* < .05 to be significant. To consider unequal selection probability and nonresponse differences, all analyses were conducted in SAS complex survey modules (version 9.3, SAS Institute Inc) by including sample weights, sampling strata, and primary sampling units in the analyses.

## Results

Of 150,760 survey participants in 23 states and the District of Columbia, 22.5% reported having a disability. Of all survey participants, 29.0% reported drinking SSB at least once daily; the prevalence of daily SSB consumption was slightly higher among adults with disabilities than among adults without disabilities (30.3% vs 28.6%, χ^2^= 7.4, *P* = .01) (Table 1). A significantly higher percentage of adults with disabilities than without disabilities were obese, female, older, had lower household income, had lower educational attainment, and were previously married. The prevalence of daily SSB intake decreased with age regardless of disability status, but it was significantly higher in adults with disabilities than in adults without disabilities in all age groups (Table 1).

**Table 1:** Sociodemographic Characteristics of Respondents, by Disability Status and Sugar-Sweetened Beverage (SSB)^a^ Consumption, Among Adults in 23 States and the District of Columbia, Behavioral Risk Factor Surveillance System, 2013

Characteristic	Adults With Disability			Adults With No Disability			*P* Value^c^
	n (%)^b^	SSB Intake, % (SE)		n (%)^b^	SSB Intake, % (SE)		
		≥Once Daily	<Once Daily		≥Once Daily	<Once Daily	
**Total respondents**	41,199 (100.0)	30.3 (0.5)	69.7 (0.5)	109,561 (100.0)	28.6 (0.3)	71.4 (0.3)	.01
**Obesity**							
No	24,287 (59.9)	30.8 (0.7)	69.2 (0.7)	81,380 (75.0)	27.3 (0.4)	72.7 (0.4)	<.001
Yes	16,912 (40.1)	29.6 (0.9)	70.4 (0.9)	28,181 (25.0)	32.3 (0.6)	67.7 (0.6)	.01
**Sex**							
Male	16,088 (47.1)	33.5 (0.9)	66.5 (0.9)	46,751 (50.8)	34.0 (0.5)	66.0 (0.5)	.63
Female	25,111 (52.9)	27.5 (0.7)	72.5 (0.7)	62,810 (49.2)	23.0 (0.4)	77.0 (0.4)	<.001
**Age, y**							
18–24	722 (5.9)	51.0 (3.6)	49.0 (3.6)	6,619 (14.3)	42.2 (1.2)	57.8 (1.2)	.02
25–34	1,813 (9.5)	45.6 (2.5)	54.4 (2.5)	12,273 (18.0)	37.2 (0.8)	62.8 (0.8)	.01
35–44	3,124 (11.2)	43.0 (1.8)	57.0 (1.8)	15,892 (17.7)	30.2 (0.7)	69.8 (0.7)	<.001
45–54	6,713 (19.6)	34.8 (1.2)	65.2 (1.2)	20,060 (18.4)	26.5 (0.7)	73.5 (0.7)	<.001
55–64	10,830 (23.6)	24.1 (0.9)	75.9 (0.9)	23,516 (15.3)	19.7 (0.6)	80.3 (0.6)	<.001
≥65	17,997 (30.2)	18.7 (0.7)	81.3 (0.7)	31,201 (16.2)	16.0 (0.5)	84.0 (0.5)	.01
**Race/ethnicity**							
Non-Hispanic white	33,314 (70.9)	28.4 (0.5)	71.6 (0.5)	88,364 (65.3)	26.2 (0.3)	73.8 (0.3)	<.001
Non-Hispanic black	4,667 (12.4)	40.3 (1.9)	59.7 (1.9)	10,687 (10.7)	39.7 (1.1)	60.3 (1.1)	.81
Hispanic	1,203 (10.0)	31.6 (3.5)	68.4 (2.5)	5,396 (15.2)	36.6 (1.1)	63.4 (1.1)	.01
Non-Hispanic other	2,015 (6.7)	29.4 (2.7)	70.6 (2.7)	5,114 (8.8)	18.7 (1.1)	81.3 (1.1)	<.001
**Annual household income, $**							
<25,000	16,382 (41.4)	35.9 (0.9)	64.1 (0.9)	20,773 (22.1)	39.0 (0.8)	61.0 (0.8)	.01
25,000 to <50,000	8,994 (20.4)	28.7 (1.2)	71.3 (1.2)	24,912 (21.8)	33.6 (0.7)	66.4 (0.7)	<.001
50,000 to <75,000	4,201 (10.4)	22.6 (1.5)	77.4 (1.5)	17,045 (14.5)	26.6 (0.8)	73.4 (0.8)	.02
≥75,000	5,806 (15.3)	20.6 (1.3)	79.4 (1.3)	33,604 (30.3)	18.0 (0.5)	82.0 (0.5)	.05
Unknown	5,816 (12.5)	32.5 (1.7)	67.5 (1.7)	13,227 (11.3)	29.3 (0.9)	70.7 (0.9)	.08
**Education level**							
<High school diploma	5,131 (20.5)	40.8 (1.6)	59.2 (1.6)	6,842 (12.4)	42.9 (1.2)	57.1 (1.2)	.31
High school diploma	13,041 (29.2)	33.6 (1.0)	66.4 (1.0)	30,282 (27.4)	36.4 (0.6)	63.6 (0.6)	.02
>High school diploma	23,027 (50.3)	24.1 (0.6)	75.9 (0.6)	72,437 (60.2)	22.1 (0.4)	77.9 (0.4)	.01
**Marital status**							
Married/couple	18,551 (49.7)	27.7 (0.8)	72.3 (0.8)	64,991 (58.6)	25.2 (0.4)	74.8 (0.4)	.01
Previously married	17,527 (32.1)	28.8 (0.8)	71.2 (0.8)	27,315 (16.5)	25.7 (0.6)	74.3 (0.6)	.01
Never married	5,121 (18.2)	40.2 (1.7)	59.8 (1.7)	17,255 (24.9)	38.5 (0.8)	61.5 (0.8)	.37

a SSBs include regular soda, fruit drink, sweet tea, and sports or energy drink.

b Unweighted sample size and weighted percentage.

c Differences in prevalence of daily SSB consumption between those with disabilities and those without disabilities determined by χ^2^ test.

Among nonobese adults, the odds of daily SSB intake were 1.27 times higher (95% CI, 1.17–1.38, *P* < .001) among adults with disabilities than among those without disabilities after controlling for sex, age, race/ethnicity, annual household income, educational attainment, and marital status (Table 2). However, among obese adults, we found no significant difference in the odds of daily SSB consumption by disability status (OR = 0.97; 95% CI, 0.86–1.09, *P* = .58). Results were the same when we further controlled for state.

**Table 2:** Adjusted^a^ Odds Ratios and 95% Confidence Intervals for Consuming Sugar-Sweetened Beverages^b^ at Least Once Daily, by Disability and Obesity Status, Among Adults in 23 States and District of Columbia, Behavioral Risk Factor Surveillance System, 2013

Variable	n	Adjusted Odds Ratio (95% Confidence Interval)	*P* Value
**Disability by obesity status**			
Not obese			
No disability	81,380	1 [Reference]	
Disability	24,287	1.27 (1.17–1.38)	<.001
Obese			
No disability	28,181	1 [Reference]	
Disability	16,912	0.97 (0.86–1.09)	.58
**Sex**			
Male	16,088	1.63 (1.54–1.72)	<.001
Female	25,111	1 [Reference]	
**Age, y**			
18–24	722	4.35 (3.83–4.94)	<.001
25–34	1,813	3.64 (3.30–4.02)	<.001
35–44	3,124	2.95 (2.69–3.23)	<.001
45–54	6,713	2.31 (2.12–2.52)	<.001
55–64	10,830	1.49 (1.36–1.62)	<.001
≥65	17,997	1 [Reference]	
**Race/ethnicity**			
Non-Hispanic white	33,314	1 [Reference]	
Non-Hispanic black	4,667	1.39 (1.27–1.52)	<.001
Hispanic	1,203	0.62 (0.54–0.71)	.001
Non-Hispanic other	2,015	0.85 (0.77–0.95)	<.001
**Annual household income, $**			
0 to <25,000	16,382	2.12 (1.93–2.33)	<.001
25,000 to <50,000	8,994	1.95 (1.78–2.12)	<.001
50,000 to <75,000	4,201	1.52 (1.38–1.67)	<.001
≥75,000	5,806	1 [Reference]	
Unknown	5,816	1.66 (1.49–1.84)	<.001
**Education level**			
<High school diploma	5,131	2.02 (1.83–2.23)	<.001
High school diploma	13,041	1.65 (1.55–1.75)	<.001
>High school diploma	23,027	1 [Reference]	
**Marital status**			
Married/couple	18,551	1 [Reference]	
Previously married	17,527	1.03 (0.96–1.10)	.44
Never married	5,121	0.95 (0.87–1.03)	.22

a Multivariable logistic regression included disability, obesity, interaction of disability and obesity and controlled for sex, age, race/ethnicity, annual household income, educational attainment, and marital status.

b Sugar-sweetened beverages include regular soda, fruit drink, sweet tea, and sports or energy drink.

## Discussion

The prevalence of consuming SSB at least once daily was 30.3% among adults with disability and 28.6% among adults with no disability in our study. The prevalence of disability for adults aged 18 years or older was 22.5% in our study, a prevalence similar to that reported in the 2010 Census Bureau, 21.3% for people aged 15 years or older, where disability was defined as having a difficulty in communicative, physical, or mental domains. The content of criteria used for each domain overlapped with the content of questions used to assess disability in the BRFSS (1).

Adults with disabilities are more likely than those without disabilities to have chronic diseases related to poor diet (5,6). However, among adults with disabilities, overall dietary intake is not well understood and the intake of foods or beverages that may contribute to poor diet quality, such as SSBs, is not documented. According to 2007–2010 NHANES data (18), the amount of saturated fat intake was likely to exceed the recommended daily limit among adults with disabilities, whereas the amount of fiber, vitamin A, vitamin C, calcium, and potassium intake was less likely to meet recommendations. Using 2011 BRFSS data, one study found that adults with disabilities consumed fruits and vegetables less frequently than adults without disabilities (22). In our study, the odds of consuming SSBs daily were 27% higher among nonobese adults with disabilities than among nonobese adults without disabilities. Because of disability-related limitations, such as severity of disabling condition, loss of appetite, and lack of physical energy, consumption of a healthy diet may be more challenging for adults with disabilities (3,4). These barriers may limit their ability to consume a healthy diet and may result in inadequate nutritional intake (eg, through consumption of processed food or fast food) (23). Eating fast food may be positively associated with increased SSB consumption among adults (24–26). One study found that among adults, fast food was associated with higher SSB intake — adults who ate fast foods drank about half of a serving more of SSBs than those who did not eat fast foods (24).

We found no significant association between disability and daily SSB intake among adults with obesity after controlling for sociodemographic factors. This finding might be due to the fact that our study was cross-sectional: adults with obesity may limit their SSB intake as a strategy for losing weight. A previous study reported that US adults who were trying to lose weight had lower SSB intake than those who were not trying to lose weight (16). Another possibility is that underlying health conditions or limitations related to obesity might have masked any associations between SSB intake and disability status.

Disability prevalence may be increasing because of advances in medical technologies (more years lived with disability), increased life expectancy, and an aging population. However, despite the nation’s progress in reducing health disparities among racial and ethnic minority groups (27,28), little attention has been given to the health disparities of people with disabilities. Identification of the factors related to poor dietary habits, including daily consumption of SSBs, among adults with disabilities can lead to strategies to improve nutrition and potentially reduce their health disparities.

Because sugar consumption increases energy intake but reduces nutritional caloric intake, the World Health Organization recommends reducing sugar intake, of which SSBs are a primary source (29). The negative affect of SSB intake on adults’ health (10–16) has not been examined exclusively among adults with disabilities, although a negative affect could be more substantial among this population than among adults without disabilities. Furthermore, identifying environmental factors associated with SSB intake among people with disabilities is needed to inform interventions to reduce SSB consumption. A healthy lifestyle includes healthy nutrition, and small lifestyle changes may especially affect the health of people with disabilities. A focus on the reduction of SSB consumption could aid in the prevention of related chronic diseases (30).

This study has several limitations. First, BRFSS data are based on self-report, which are subject to recall and reporting bias. Second, SSB intake was measured as a frequency in a food-frequency questionnaire (FFQ) rather than as an amount or as calories. However, although a direct comparison cannot be made with our study, a previous study found that estimates of beverage intake from a fully quantitative FFQ were similar to estimates from a 24-hour dietary recall (31). Third, because only 23 states and the District of Columbia participated in the optional BRFSS Sugar Drinks Module*,* our findings may not be generalizable to the entire US adult population. Fourth, the BRFSS disability questions do not assess disability severity and type or distinguish between mental and physical disabilities; these factors could influence beverage choices among adults with disabilities. For example, adults with severe disability might receive complete diet care from their caregivers, and the accurate report from people with mental disabilities can be difficult; such factors may confound the associations examined in our study. Fifth, about 13% of participants had unknown household income but were still included in the analysis so that we would not lose information on the main variables (ie, disability, SSB consumption, and obesity). Sixth, survey participants who use cellular telephones exclusively may have different characteristics than participants who do not, which may limit their representativeness. Finally, BRFSS data are cross-sectional, therefore, causation and directionality of association between SSB intake and disability by obesity status cannot be determined.

Our study found that 3 of 10 adults with disability consumed an SSB at least once daily. The prevalence of daily SSB consumption among adults with disabilities was slightly higher than among adults without disabilities; however, because this was one cross-sectional study, we do not know whether this difference is meaningfully significant. Moreover, nonobese adults with disability had higher odds of consuming an SSB at least once daily than nonobese adults without disability after controlling for sociodemographic factors. Our findings suggest that there is a need to increase awareness of SSB intake among adults with disabilities, because, given their possible limited mobility and other health conditions, adults with disabilities may be at even higher risk of developing chronic diseases than their counterparts. Health promotion program practitioners should be aware of the high prevalence of daily SSB intake in this population. Targeted intervention strategies may increase awareness that an unhealthy diet, consisting of frequent SSB intake, is associated with adverse health consequences. Finally, more research is needed to assess the effect of frequent SSB consumption on the increased chronic health risks among adults with disabilities.
